# Maternal outcomes following prenatal spina bifida repair: A systematic review and meta‐analysis

**DOI:** 10.1002/pmf2.70230

**Published:** 2026-01-19

**Authors:** Nikan Zargarzadeh, Ehsan Rojhani, Ali Javinani, May Abiad, Enaja Sambatur, Eyal Krispin, Ashish Premkumar, Ramen H. Chmait, Ramesha Papanna, Jena Lyn Miller, Kjersti Aagaard, Alireza A. Shamshirsaz

**Affiliations:** ^1^ Fetal Care and Surgery Center (FCSC) Division of Fetal Medicine and Surgery Boston Children's Hospital, Harvard Medical School Boston Massachusetts USA; ^2^ Department of Obstetrics and Gynecology George Washington University Hospital Washington District of Columbia USA; ^3^ Department of Obstetrics and Gynecology Baptist Medical Group, Baptist Memorial Health Care Memphis Tennessee USA; ^4^ Department of Obstetrics and Gynecology Section of Maternal Fetal Medicine Pritzker School of Medicine at the University of Chicago Chicago Illinois USA; ^5^ Division of Maternal‐Fetal Medicine Department of Obstetrics and Gynecology Keck School of Medicine, University of Southern California Los Angeles California USA; ^6^ Division of Fetal Intervention Department of Obstetrics Gynecology and Reproductive Sciences McGovern Medical School, University of Texas Health Science Center Houston Texas USA; ^7^ Johns Hopkins Center for Fetal Therapy Department of Gynecology and Obstetrics Johns Hopkins University School of Medicine Baltimore Maryland USA; ^8^ Department of Obstetrics and Gynecology HCA Healthcare and HCA Healthcare Research Institute Nashville Tennessee USA; ^9^ Department of Obstetrics and Gynecology HCA Texas Maternal Fetal Medicine Houston Texas USA

**Keywords:** fetal surgery, meta‐analysis, maternal complications, neural tube defect

## Abstract

**Objective:**

Prenatal surgical interventions for neural tube defects have been shown to improve fetal outcomes but are associated with previously poorly quantified maternal risk, which is inherently dependent on the surgical approach used. We hypothesized that a meta‐analysis would enable objective quantitation of maternal risk and safety, stratified by which antenatal neural tube defect surgical approaches were employed.

**Study design:**

We conducted a meta‐analysis by searching PubMed, Scopus, Embase, and Web of Science databases. We included all studies that performed prenatal intrauterine neural tube defect repair, regardless of the surgical technique. Our primary safety measure outcomes were maternal mortality and severe maternal morbidity (inclusive of uterine rupture or dehiscence, blood transfusion, and pulmonary edema). A total of 26 studies published between 2010 and 2024 were selected, prioritizing larger sample sizes and relevant outcomes when populations overlapped. The meta‐analysis was conducted using random‐effects models to account for between‐study heterogeneity, which was quantified using the *I* ^2^ statistic; sensitivity analyses and meta‐regression were performed to assess robustness and explore sources of heterogeneity. Analyses were conducted using R software (version 4.0.5).

**Results:**

Our meta‐analysis included a total of 1485 patients. No occurrence of maternal mortality was reported in any included studies (0/995). Some studies provided comprehensive data for all complications, while others only reported specific outcomes, leading to variation in case denominators: uterine rupture or dehiscence was observed in a pooled proportion of 4% (95% confidence interval [CI], 1%–11%), occurring in 7.4% of open surgery cases (53/710), 3.1% of mini‐hysterotomy cases (6/192), and 0% of fetoscopic cases (0/157). Among 872 reported cases, maternal pulmonary edema occurred in 3% (pooled estimate: 0.03; 95% CI, 0.02–0.06). The lowest rate of pulmonary edema was seen with open surgical approaches (20 of 661 cases, 3%), followed by mini‐hysterotomy (2 of 55 cases, 3.6%) and fetoscopic surgeries (15 of 159 cases, 9.4%). Model‐adjusted pooled proportions demonstrated an overall low rate of maternal blood transfusion at 0.03 (95% CI, 0.01–0.05) among 971 cases.

**Conclusion:**

This study highlights the need for standardized and comprehensive reporting of maternal outcomes in fetal surgery in order to reliably counsel patients on anticipated and acceptable maternal risk. However, despite limited data, our findings suggest that maternal mortality is rare and that severe maternal complications occur infrequently; however, the potential for serious morbidity underscores the need for careful patient selection, counselling, and standardized outcome reporting.

## 1 INTRODUCTION

Prenatal surgical repair of open neural tube defects (NTDs), such as myelomeningocele (spina bifida), has transformed the prognosis for affected infants but poses recognized, albeit poorly quantitated, risks to the gravida [1]. The landmark Management of Myelomeningocele Study (MOMS) demonstrated that in utero repair via open hysterotomy significantly improves infant outcomes—reducing hindbrain herniation and shunt dependence and improving motor function—compared to postnatal repair [2]. However, this fetal benefit was accompanied by known risks of obstetric complications, including preterm labor, prelabor rupture of membranes, placental abruption, and surgical‐site morbidities [3]. In MOMS and subsequent series, maternal near‐miss events (life‐threatening complications short of mortality) were infrequent but notable—for example, pulmonary edema occurred in ∼6% of cases, significant hemorrhage requiring transfusion in ∼9%, and roughly one‐third of mothers had uterine scar thinning or dehiscence at a subsequent delivery [4]. Of further concern, isolated cases of frank uterine rupture have been reported at a rate of 1%–2% in large cohorts, underscoring the potential for catastrophic outcomes in both the index and subsequent pregnancies [4]. Importantly, although no maternal deaths have been reported in modern fetal surgery series, the possibility of maternal “near‐miss” morbidity necessitates rigorous and evidence‐based patient counselling and monitoring [5]. The fundamental challenge is the absence of data providing rigorous collective quantitation of maternal risk.

In response to these risks, the field has advanced surgical approaches aimed at improving maternal safety and emphasized standardized reporting of complications to enable comparative risk estimates. Fetoscopic repairs (either entirely percutaneous endoscopic or laparotomy‐assisted “hybrid” approaches) avoid a large hysterotomy and have shown the potential to reduce maternal abdominal wall incision size, reduce uterine hysterotomy rate, reduce blood loss, and enable vaginal birth while conferring similar fetal benefits [5, 6, 7, 8]. These potential risk reductions were not without counterbalanced elevated risk, including risk of preterm premature rupture of membranes and prolonged operative time with ensuing elevated risk of maternal pulmonary edema. Recognizing the absence of a comprehensive synthesis of maternal risk across prenatal NTD repair techniques, the primary aim of this study was to systematically evaluate maternal outcomes following fetal surgery. Specifically, we sought to compare maternal morbidity across surgical approaches to provide a more robust evidence base for patient counselling. Our second study aim was to recognize gaps and disparities in maternal outcome reporting and generate guidance for uniform reporting in future clinical trials and prospective studies.

## 2 METHODS

### 2.1 Study design and registration

This systematic review and meta‐analysis adhered to the PRISMA (Preferred Reporting Items for Systematic Reviews and Meta‐Analyses) 2020 guidelines to ensure transparency and rigor. The primary objective was to assess maternal outcomes following prenatal surgical repair of NTDs, with a focus on safety and without direct comparison of different surgical techniques.

### 2.2 Eligibility criteria

The inclusion criteria for this meta‐analysis were defined using the Population, Intervention, Comparison, and Outcome (PICO) framework:

- *Population*: Pregnant women (gravidae) diagnosed with NTDs in the fetus who underwent prenatal surgical repair.
- *Intervention*: Any form of prenatal surgical intervention for NTD repair, including approaches such as open repair, mini‐hysterotomy, laparotomy‐assisted fetoscopic repair, and percutaneous fetoscopic repair.
- *Comparison*: Maternal outcomes are reported according to surgical approach (open repair, mini‐hysterotomy, laparotomy‐assisted fetoscopic repair, and percutaneous fetoscopic repair).
- *Outcomes*: The outcomes of interest were defined a priori. The primary outcomes were maternal mortality and severe maternal morbidity, specifically uterine rupture or dehiscence, pulmonary edema, and blood transfusion. The secondary outcomes included chorioamnionitis, placental abruption, oligohydramnios, membrane separation, uterine atony, and pulmonary embolism.

Eligible studies were randomized controlled trials (RCTs) or observational studies (cohort, case‐control, or case series) with more than five cases that reported maternal outcomes related to prenatal NTD repair. Exclusion criteria included systematic reviews, case reports, conference abstracts, and studies with insufficient or mixed outcome data without stratification.

### 2.3 Search strategy

A comprehensive literature search was conducted across four major databases: PubMed, Embase, Scopus, and Web of Science. This search included studies published between May 2009 and January 2025. The search strategy employed a combination of relevant keywords and Medical Subject Headings (MeSH) terms, such as “Prenatal Surgery,” “Neural Tube Defects,” “Maternal Outcomes,” and “Surgical Techniques.” Detailed search terms and filters are provided in the Supporting Information. In addition to database searches, reference lists from key studies were manually reviewed to identify any additional relevant articles. Titles and abstracts were independently screened by two reviewers (E.R. and E.S.) using Rayyan software, with discrepancies resolved through discussion or consultation with a third reviewer (N.Z.). The full‐text screening was similarly conducted independently by the two reviewers, with any conflicts addressed collaboratively.

### 2.4 Data extraction and management

Data extraction was performed using a standardized form, which was pilot tested to ensure consistency. Extracted data included study characteristics (e.g., first author, publication year, country, and study design) and demographic information (e.g., maternal age, body mass index [BMI], hypertensive disorders of pregnancy [HTN], gestational diabetes mellitus [GDM], and gestational age at intervention). Outcomes designated as primary were maternal mortality and major maternal morbidity, including uterine rupture or dehiscence, pulmonary edema, and the need for blood transfusion. Outcomes designated as secondary were chorioamnionitis, placental abruption, oligohydramnios, membrane separation, uterine atony, and pulmonary embolism. To ensure accuracy, two reviewers independently extracted data, with any discrepancies resolved through discussion or by involving a third reviewer. In cases where studies reported overlapping patient populations, priority was given to studies with the largest sample sizes or the most detailed outcome reporting. Definitions of outcomes (e.g., uterine rupture, uterine atony, pulmonary edema) were based on those provided by each included study, as no standardized criteria were applied across all studies.

### 2.5 Quality assessment

The quality of included studies was assessed using the Newcastle‐Ottawa Scale (NOS) for observational studies, evaluating selection, comparability, and outcome assessment. For RCTs, the Cochrane Risk of Bias (RoB 2.0) tool was employed, assessing biases related to randomization, adherence, outcome measurement, and reporting. Quality assessments were performed independently by two reviewers, with disagreements resolved through discussion.

### 2.6 Statistical analysis

Statistical analyses were conducted using R software (version 4.0.5) to ensure robust and reproducible results. For continuous outcomes, pooled means and standard deviations were calculated using the inverse‐variance method. Dichotomous outcomes were synthesized using the generalized linear mixed model (GLMM) to compute pooled proportions with 95% confidence intervals (CIs). Given the expected variability across studies, random‐effects models were applied to account for heterogeneity in study designs, populations, and outcome measurements. Heterogeneity was assessed using the *I* ^2^ statistic, with thresholds set as follows: low heterogeneity (*I* ^2^ < 25%), moderate heterogeneity (25% ≤ *I* ^2^ < 75%), and high heterogeneity (*I* ^2^ ≥ 75%). To explore sources of heterogeneity, meta‐regression and subgroup analyses were performed. Sensitivity analyses, including leave‐one‐out analysis, were conducted to evaluate the impact of individual studies on overall estimates and heterogeneity. Publication bias was assessed using Egger's test and visually through funnel plots.

## 3 RESULTS

### 3.1 Study characteristics

**FIGURE 1:**
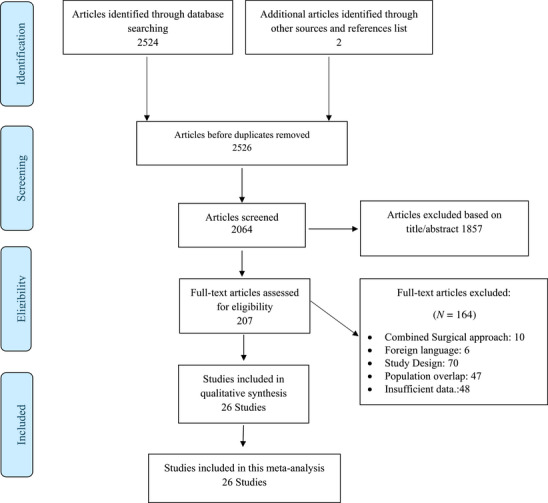
Flow diagram of the study selection process.

We included 26 studies reporting maternal outcomes following prenatal repair of NTDs, comprising a total of 1485 cases [2, 7, 9, 10, 11, 12, 13, 14, 15, 16, 17, 18, 19, 20, 21, 22, 23, 24, 25, 26, 27, 28, 29, 30, 31, 32]. The study selection process is illustrated in Figure 1, and the characteristics of the included studies—including surgical techniques and approaches alongside patient demographics—are summarized in Table 1. Risk of bias assessment is provided in the Supporting Information.

**TABLE 1:** Articles population.

Study population ID^a^	First author	Year of publication	Institution	Method of surgery	Case numbers
1	Eyal Krispin	2023	USA, Texas Children's Fetal Center	Laparotomy‐assisted fetoscopic	102
2	Chloe Arthuis	2022	France, Necker–Enfants Malades Hospital	Laparotomy‐assisted fetoscopic	7
3	Cleisson F. A. Peralta	2020	Brazil, Heart & at Pro Matre Maternity Hospital	Mini‐hysterotomy	176
4	Edgardo Corral	2019	Chile, Hospital Regional Rancagua	Mini‐hysterotomy	16
5	Luana Sarmento Neves da Rocha	2021	Brazil, Hospital das Clínicas da Faculdade (HCFMUSP)	Mini‐hysterotomy	39
6	Eyal Krispin	2023	USA, Texas Children's Fetal Center	Open repair	44
7	Julie S. Moldenhauer	2014	USA, CHOP	Open repair	100
8	Mateusz Zamły´nski	2023	Poland, Fetal Surgery Center Bytom	Open repair	60
8	Tomasz Horzelski	2023	Poland, Bytom, Medical University of Silesia, Katowice	Open repair	57
9	Ueli Moehrlen	2021	Switzerland, Zurich	Open repair	148
10	Matthew E. Pontell	2023	USA, Vanderbilt	Open repair	22
11	Matthew E. Pontell	2023	USA, Vanderbilt	Open repair	64
12	Vagisha Pruthi	2020	Canada, Ontario Fetal Centre	Open repair	27
13	Smruti K. Patel	2023	USA, Cincinnati Fetal Center	Open repair	56
13	Stefanie Riddle	2019	USA, Cincinnati Fetal Center	Open repair	23
14	Adzick Scot	2011	USA, (CHOP; Vanderbilt; UCSF)	Open repair	78
14	Mark P. Johnson	2016	CHOP, Vanderbilt, UCSF	Open repair	91
15	Jacek Zamły´nski´	2014	Poland, Bytom	Open repair	46
16	AF Moron	2018	Brazil, Maternidade Santa Joana	Open repair	237
17	Rogelio Cruz‐Martinez	2021	Mexico, Queretaro	Open repair	13
18	Guilbaud Lucie	2021	France, Trousseau Hospital	Open repair	17
19	Kanokwaroon Watananirun	2024	Belgium UZ Leuven, Brazil Heart Hospital of Sao Paulo	Open repair	78
20	Wagner Jou Hisaba	2012	Brazil, Federal University of São Paulo (UNIFESP)	Open repair	6
21	C.D. Goonasekera	2020	England, King's College hospital	Percutaneous fetoscopic	5
22	Giorgio Carrabba	2019	Italy, Fondazione IRCCS Ca’ Granda Ospedale Maggiore	Percutaneous fetoscopic	5
23	D. A. Lapa	2018	Brazil, Servidor Publico Estadual, Samaritano, Albert Einstein	Percutaneous fetoscopic	45
24	Miriam Ziemann	2018	Germany, DZFT University of Giessen	Percutaneous fetoscopic	65
24	Thomas Kohl`	2010	Germany, University of Bonn	Percutaneous fetoscopic	16

^a^ Studies sharing the same population ID were considered overlapping; therefore, only one set of outcomes from each population was included in the analysis to avoid duplication.

These studies, published between May 2009 and 2024, describe comparative outcomes for open repair, mini‐hysterotomy, laparotomy‐assisted fetoscopic repair, and percutaneous fetoscopic repair. In cases of overlapping index study reporting, only the primary study report with the most complete and relevant outcome data was retained in our analysis, thereby eliminating duplication of reporting for any single subject or study participant. The sample sizes presented in each outcome reflect the number of study participants with available data. Detailed information and all pooled outcome data are summarized in Table 2, and the corresponding forest plots are available in the Supporting Information.

**TABLE 2:** Primary and secondary outcomes.

Outcome	Studies	Total cases	Events	Pooled proportion/pooled mean	95% CI	Heterogeneity (*I* ^2^)
Maternal mortality	12	995	0	0	[0.00; 1.00]	0%
Pulmonary edema	14	872	–	0.03	[0.03; 0.06]	35%
Uterine rupture/dehiscence	14	1048	–	0.04	[0.01; 0.11]	78%
Blood transfusion	14	971	–	0.03	[0.01; 0.05]	15%
Chorioamnionitis	14	950	–	0.05	[0.02; 0.1]	76%
Membrane separation	10	744	–	0.21	[0.12; 0.32]	76%
Oligohydramnios	14	861	–	0.17	[0.1; 0.27]	72%
Placental abruption	17	1268	–	0.03	[0.02; 0.06]	55%
Uterine atony	4	201	2	0	[0; 0.81]	0%
Embolism	6	438	–	0.01	[0; 0.04]	0%
HTN of pregnancy	9	476	–	0.03	[0.02; 0.06]	0%
Gestational diabetes mellitus (GDM)	7	403	–	0.04	[0.02; 0.12]	49%
Mean maternal age	18	1186	–	29.94	[29.32; 30.55]	70%
Mean maternal BMI	15	889	–	26.3	[25.60; 27.01]	73%
Mean GA at birth	22	1275	–	34.23	[33.62; 34.84]	92%
Mean GA at intervention	24	1337	–	25.01	[24.48; 25.55]	98%
Mean duration of surgery	18	1172	–	162.24	[138.85; 185.64]	100%

Abbreviations: BMI, body mass index; CI, confidence interval; GA, gestational age; HTN, hypertension.

### 3.2 Maternal outcomes

Maternal mortality was reported in 12 studies encompassing 995 cases. No maternal deaths were reported with no observed heterogeneity (*I* ^2^ = 0%). Uterine rupture or dehiscence was observed in 4% of 1048 cases (95% CI, 1%–11%), including 7.4% in open surgery, 3.1% in mini‐hysterotomy, and 0% in fetoscopic cases.

**FIGURE 2:**
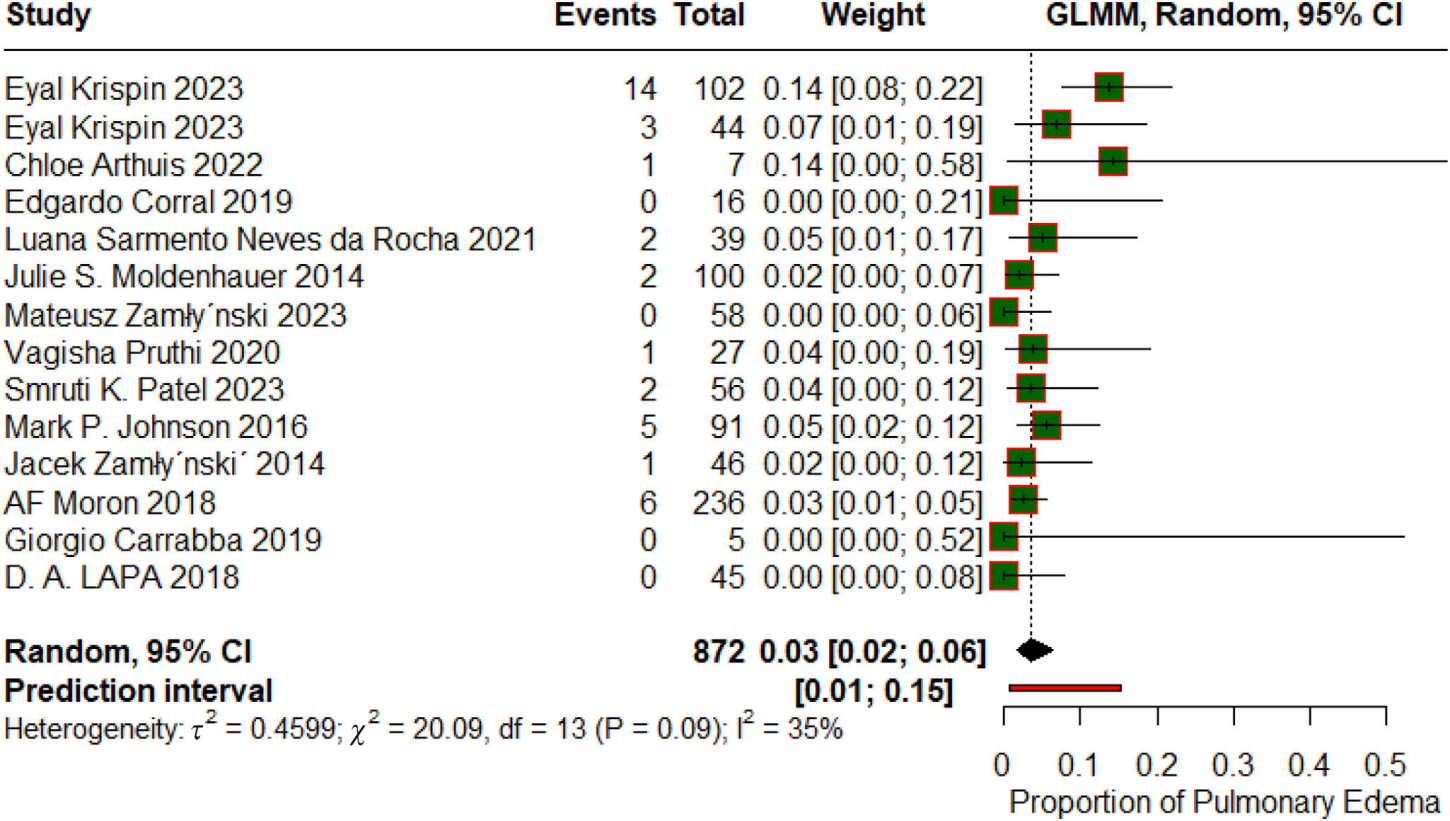
Pulmonary edema. CI, confidence interval; GLMM, generalized linear mixed model.

Pulmonary edema was reported in 14 studies encompassing 872 cases, with a pooled incidence of 3% (95% CI, 2%–6%) and moderate heterogeneity (*I* ^2^ = 35%) (3% of cases [20/661] undergoing open surgery, 3.6% of cases [2/55] undergoing mini‐hysterotomy, and 9.4% of cases [15/159] undergoing fetoscopic surgery) (Figure 2). One study was identified as a statistical outlier; when excluded, heterogeneity dropped to 0%, while the pooled estimate remained unchanged. Preliminary meta‐Pontellion suggested that pulmonary edema was marginally associated with longer surgical duration (*p* = 0.05) and inversely associated with maternal age (*p* = 0.01).

Blood transfusion was reported in 14 studies (971 cases), with a pooled incidence of 3% (95% CI, 1%–5%) (3.5% of cases [21/595] undergoing open surgery, 2.2% of cases [5/231] undergoing mini‐hysterotomy, and 2.5% of cases [4/159] undergoing fetoscopic surgery).

Among maternal complications, chorioamnionitis was most frequently reported with a pooled incidence of 5% (95% CI, 2%–10%). Placental abruption occurred in 3% of 1268 cases across 17 studies (95% CI, 2%–6%). Uterine atony, reported in four studies (201 cases), resulted in only two events, with a pooled estimate of 0.00% (95% CI, 0.00–0.81). Pulmonary embolism, reported in six studies (438 cases), had a pooled proportion of 1% (95% CI, 0.00%–4%).

### 3.3 Heterogeneity and sensitivity analysis

Outlier analysis and leave‐one‐out sensitivity analysis confirmed the robustness of the estimate, with no individual study substantially affecting the overall result. Publication bias assessment using Egger's test indicated potential asymmetry prior to outlier exclusion (*p* = 0.0486), which resolved after removal (*p* = 0.81). All related sensitivity and funnel plots are provided in the Supporting Information.

## 4 DISCUSSION

This meta‐analysis enabled us to report relatively rare maternal outcomes following prenatal surgical repair for spina bifida. Among 995 analyzed cases, no maternal deaths occurred. Observed severe maternal complications included pulmonary edema (3%), uterine rupture or dehiscence (4%), blood transfusion (3%), chorioamnionitis (5%), and placental abruption (3%), although study heterogeneity varied. Rare complications such as uterine atony (0%) and pulmonary embolism (1%) exhibited minimal variability due to their rarity of occurrence. Meta‐regression identified longer surgical duration as a significant risk factor for pulmonary edema.

Severe maternal morbidity warrants close attention [33, 34]. Given the inherent maternal risks with no direct maternal benefit that accompanies fetal surgical procedures, it is important to accurately quantify risks to enable informed decision‐making [35, 36, 37]. This priority aligns with findings from a 2019 systematic review, which reported maternal complication rates of 6.2% for fetoscopic surgeries and 20.9% for open fetal surgeries. Severe maternal complications occurred in 1.7% and 4.5% of these cases, respectively [5]. The Clavien‐Dindo classification, a standardized system for grading surgical complications based on severity and required interventions [38], has been adapted in various forms to evaluate maternal complications in prenatal surgery [5, 7]. Krispin et al. highlighted fewer Grade III complications and a lower Comprehensive Complication Index (CCI) with fetoscopic approaches compared to open hysterotomy [7]. Similarly, the International Fetoscopic Spina Bifida Repair Consortium reported on maternal morbidity, highlighting differences in complication rates between percutaneous and laparotomy‐assisted fetoscopic methods [39]. Vonzun et al. similarly found that although 84% of gravidae undergoing fetoscopic myelomeningocele repair experienced complications, only 6% faced life‐threatening Grade IV complications, with no maternal deaths [40]. Consistent with these observations, our analysis found no cases of pulmonary edema or uterine rupture/dehiscence in the fetoscopic group; however, the limited number of studies reporting on these outcomes constrained the robustness of our conclusions and limited our confidence. Notably, not all studies report significant safety differences between open and fetoscopic approaches. Mikulski et al. found comparable maternal complication rates between these techniques, with fetoscopic methods associated with slightly elevated intrauterine infection rates (chorioamnionitis, ∼4%) [41]. The North American Fetal Therapy Network (NAFTNet) registry offers additional context, analyzing 1213 cases between 2011 and 2024. The NAFTNet data revealed findings closely aligned with ours, with comparable rates of pulmonary edema (1.5%), placental abruption (4.5%), chorioamnionitis (5.4%), and blood transfusions (1.1%). Despite increased morbidity, both studies affirm no maternal deaths and preservation of ventriculoperitoneal shunt rate reductions, even with very preterm deliveries [42].

The higher observed rate of pulmonary edema in fetoscopic procedures should be interpreted cautiously, as it may reflect selection bias, center‐level practice variation, or differential reporting rather than an intrinsic procedural risk. Inconsistent outcome denominators and selective reporting across studies further contribute to uncertainty and limit direct comparability of pooled estimates. While fetal surgery continues to improve fetal outcomes, our study highlights a persistent gap in the literature: the underreporting and inconsistent classification of maternal complications. Most published studies emphasize fetal benefits [43], with maternal morbidity either insufficiently detailed or lacking structured categorization. Although our pooled estimates showed relative homogeneity across studies, heterogeneity in outcome definitions and underreporting limit interpretability and highlight the need for greater consistency in maternal outcome reporting. To improve consistency and clinical relevance in the reporting of maternal complications associated with fetal surgery, adopted use of standardized classification frameworks is recommended. This implementation would enhance transparency and facilitate cross‐study comparability, thereby supporting evidence‐based counselling and clinical decision‐making. Maternal outcomes beyond the perioperative period also warrant greater attention.

In addition, obtaining truly informed consent in fetal surgery presents unique ethical challenges, as parents are often highly motivated to pursue any intervention perceived to benefit the fetus [44]. This context underscores the importance of transparent risk communication, standardized outcome reporting, and multidisciplinary counselling to support informed, value‐based decision‐making.

Building on these observations, a structured, consensus‐based reporting framework specific to fetal surgery may help address current inconsistencies in maternal outcome reporting. Analogous to PRISMA for systematic reviews or strengthening the reporting of observational studies in epidemiology for observational studies, such a framework could define a core maternal outcome set, standardized severity grading, attribution to fetal intervention, perioperative and longitudinal follow‐up intervals, and minimum reporting standards for adverse events [45]. Development of this framework through multidisciplinary expert consensus would promote transparency and comparability across studies, while journal endorsement could further support consistent implementation and improve the quality of evidence synthesis in fetal surgery research.

Most included studies involved relatively healthy women at baseline, consistent with MOMS trial eligibility (younger age, normal BMI, and the absence of major comorbidities). As surgical criteria broaden to higher‐risk populations, operative safety must be carefully evaluated. In addition, the maternal consequences of postoperative bed rest and lifestyle restrictions associated with open fetal surgery have not been systematically assessed. Long‐term maternal health remains insufficiently investigated. Although studies such as Goodnight et al. [46] and Haenen et al. [47] have described obstetric and reproductive outcomes after open maternal‐fetal surgery, data on fertility, gynecologic sequelae, and psychological well‐being are scarce. Because open fetal surgery requires a high uterine incision similar to a classical caesarean, the risks of rupture, dehiscence, and abnormal placentation, including placenta accreta spectrum (PAS), in later pregnancies must be considered during preoperative counselling [46, 48]. Recommendations regarding interpregnancy interval, planned caesarean delivery, and antepartum monitoring further underscore the need for systematic, long‐term maternal follow‐up.

In conclusion, this meta‐analysis provides pooled estimates of maternal complications following prenatal surgery for spina bifida and highlights important evidence gaps. While advances in minimally invasive techniques have contributed to improved safety profiles, variation in outcome definitions and incomplete reporting across studies limit comparability. Greater adoption of standardized classification frameworks for maternal outcomes would strengthen transparency, enable cross‐study synthesis, and support more robust counselling and clinical decision‐making.

## CONFLICT OF INTEREST STATEMENT

None of the authors have a conflict of interest to disclose.

## FUNDING INFORMATION

The authors received no specific funding for this work.

## Supporting information



Supporting Information
