# Supplementary material for: Maternal outcomes following prenatal spina bifida repair: A systematic review and meta‐analysis
Source: Pregnancy (Hoboken). 2026 Jan 19;2(1):e70230. doi: 10.1002/pmf2.70230 (PMC13344694; doi:10.1002/pmf2.70230)
Supplement: Supplementary file 1 [file PMF2-2-e70230-s001.docx]

Table 1: Quality Assessment

Figure 1: Maternal age funnel plot

Figure 2: Maternal BMI funnel plot

Figure 3: Gestational diabetes mellitus (GDM) funnel plot

Figure 4: Hypertensive disorders of pregnancy (HDP) funnel plot

Figure 5: Duration of surgery funnel plot

Figure 6: Gestational age at birth funnel plot

Figure 7: Maternal Mortality funnel plot

Figure 8: Uterine rupture and dehiscence funnel plot

Figure 9: Blood transfusion funnel plot

Figure 10: Chorioamnionitis funnel plot

Figure 11: Placental abruption funnel plot

Figure 12: Uterine atony

Figure 13: Leave-One-Out Sensitivity Analysis

Figure 14: Outlier Detection

Meta-Regression

Publication Bias Assessment

**Table 1. Risk of Bias assessment. (Quality Assessment)**

| Study ID | First Author | Selection (one star per question) | | | | Comparability (maximum two stars) | Outcome (one star per question) | | | Total |
| --- | --- | --- | --- | --- | --- | --- | --- | --- | --- | --- |
|  |  | Representativeness | Non-exposed | Ascertainment | Not present at start of study |  | Assessment of outcome | Was follow-up long enough for outcomes to occur | Adequacy of follow-up of cohorts |  |
| 62 | Eyal Krispin | * | * | * | * | ** | * | * | * | 9 |
| 80 | Cleisson F. A. Peralta | * |  | * | * |  | * | * | * | 6 |
| 73 | Ueli Moehrlen | * |  | * | * |  | * | * | * | 6 |
| 82 | Matthew E. Pontell | * | * | * | * | ** | * | * | * | 9 |
| 74 | Julie S. Moldenhauer | * |  | * | * |  | * | * | * | 6 |
| 83 | Vagisha Pruthi | * |  | * | * |  | * | * | * | 6 |
| 84 | Stefanie Riddle | * | * | * | * | * | * | * | * | 8 |
| 112 | Jacek Zamły´nski´ | * | * | * | * | ** | * | * | * | 9 |
| 113 | Mateusz Zamły´nski | * |  | * | * |  | * | * | * | 6 |
| 117 | Miriam Ziemann | * |  | * | * |  | * | * | * | 6 |
| 5 | Chloe Arthuis | * |  | * | * |  | * | * | * | 6 |
| 11 | AF Moron | * | * | * | * | ** | * | * | * | 9 |
| 16 | Cara Buskmiller | * |  | * | * |  | * | * | * | 6 |
| 17 | Giorgio Carrabba | * |  | * | * |  | * | * | * | 6 |
| 22 | Edgardo Corral | * |  | * | * | * | * | * | * | 7 |
| 34 | Luana Sarmento Neves da Rocha | * |  | * | * |  | * | * | * | 6 |
| 46 | C.D. Goonasekera | * |  | * | * |  | * | * | * | 6 |
| 48 | Guilbaud Lucie | * | * | * | * | ** | * | * | * | 9 |
| 51 | Wagner Jou Hisaba | * |  | * | * |  | * | * | * | 6 |
| 52 | Tomasz Horzelski | * | * | * | * | ** | * | * | * | 9 |
| 60 | Thomas Kohl` | * |  | * | * |  | * | * | * | 6 |
| 101 | Noel Tulipan | * | * | * | * | ** | * | * | * | 9 |
| 118 | Adzick Scott | * | * | * | * | ** | * | * | * | 9 |
| 56 | Mark P. Johnson | * | * | * | * | ** | * | * | * | 9 |
| 119 | Kanokwaroon Watananirun | * | * | * | * | ** | * | * | * | 9 |
| 120 | Smruti K. Patel | * |  | * | * |  | * | * | * | 6 |

**Figure 1: Maternal age funnel plot**

**Figure 2: Maternal BMI funnel plot**

**Figure 3: Gestational diabetes mellitus (GDM) funnel plot**

**Figure 4: Hypertensive disorder of pregnancy (HDP) funnel plot**

**Figure 5: Duration of surgery funnel plot**

**Figure 6: Gestational age at birth funnel plot**

**Figure 7: Maternal Mortality funnel plot**

**Figure 8: Uterine rupture and dehiscence funnel plot**

**Figure 9: Blood transfusion funnel plot**

**Figure 10: Chorioamnionitis funnel plot**

**Figure 11: Placental abruption funnel plot**

**Figure 12: Uterine atony**

**Figure 13: Leave-One-Out Sensitivity Analysis**

**Leave-One-Out Sensitivity Analysis**: The leave-one-out analysis further confirmed the robustness of the results, as excluding any single study did not substantially change the overall proportion of pulmonary edema

**Figure 14: Outlier Detection**

**Outlier Detection**: One outlier study was identified ("Eyal Krispin 2023"). Removing this outlier reduced the heterogeneity to I^2^= 0.0% and led to a more precise estimate (proportion = 0.0299 [95% CI: 0.0190, 0.0466]).

**Meta-Regression**

The clinical interpretation of this study suggests that both maternal age and surgical duration are relevant factors in the occurrence of pulmonary edema. Meta-regression analysis showed a marginal association between longer surgery duration and increased risk of pulmonary edema (p = 0.0496), while increasing maternal age was significantly associated with a reduced risk (p = 0.0100). However, this apparent protective effect of maternal age may be confounded by surgical technique, as older patients were more likely to undergo open repair—a method typically associated with shorter operative times. Due to the strong inverse correlation between surgical duration and open technique (r = –0.755), surgical technique was excluded from the multivariable model to avoid multicollinearity.

Multimodel inference using AICc identified both maternal age and surgical duration as top-ranking predictors, and the final model showed no evidence of multicollinearity. However, permutation testing attenuated the statistical significance of both variables, suggesting that these findings may reflect random variation rather than true causal relationships. As such, while the results highlight potentially important trends, they should be interpreted cautiously and viewed as hypothesis-generating rather than definitive evidence. Gestational age at intervention and surgical technique were not statistically significant in either univariate or multivariable models. The best-fitting model included both "Duration of Surgery" and "Maternal Age" as predictors, highlighting their importance in explaining the variability in pulmonary edema proportions. The "Open Repair" surgery technique had a lower importance score in model selection, indicating it was less influential in predicting pulmonary edema compared to other factors.

#### 1. **Multi-Collinearity Assessment**

- **Correlation Analysis**: The correlation between predictors (e.g., "Duration of Surgery" and "Maternal Age") was assessed to check for multi-collinearity. The analysis showed moderate correlations, with no severe multi-collinearity observed (correlation coefficients < |0.8|).

The correlation matrix provides insight into the relationships between the variables in your model. Here is what each value means: Duration of surgery vs. Maternal age: r=0.137 this is a weak positive correlation. Duration of surgery vs. Open repair: r=−0.755 This is a strong negative correlation, indicating that as the duration of surgery increases, the likelihood of using the "Open repair" technique decreases, or vice versa. Maternal age vs. Open repair: r=−0.502, this is a moderate negative correlation. The strong negative correlation between "Duration of surgery “and "Open repair" raises a concern for multi-collinearity. In regression models, high correlation (typically 𝑟>0.7 or r<−0.7) between predictors can lead to issues where the model struggles to distinguish the independent effects of these variables. So we removed one of them (Open repair) from multiregression.

**Publication bias assessment**

- **Egger's Test**: Before removing outliers: t=−2.20t = -2.20t=−2.20, p=0.0486p = 0.0486p=0.0486 (indicating potential publication bias). After removing outliers: t=−0.24t = -0.24t=−0.24, p=0.8138p = 0.8138p=0.8138 (no evidence of publication bias).
- **Begg's Test**: z=0.41z = 0.41z=0.41, p=0.6808p = 0.6808p=0.6808 (no evidence of publication bias).
- **Trim and Fill Method**: Adjusted proportion after filling: 0.0360 [95% CI: 0.0248, 0.0521], confirming the robustness of the overall estimate despite potential biases.
